# Effect of Multiple Stresses (Thermal, Nutritional, and Walking Stress) on the Reproductive Performance of Malpura Ewes

**DOI:** 10.1155/2012/471760

**Published:** 2012-02-09

**Authors:** V. Sejian, V. P. Maurya, K. Kumar, S. M. K. Naqvi

**Affiliations:** Adaptation Physiology Laboratory, Division of Physiology and Biochemistry, Central Sheep and Wool Research Institute, Avikanagar, Rajasthan 304501, India

## Abstract

A study was conducted to assess the effect of multiple stresses (thermal, nutritional, and walking stress) on the reproductive performance of Malpura ewes. The study was conducted for a period of 35 days covering two estrous cycles during summer season (April-May). The ewes were randomly allocated into two groups of twelve animals each namely, GI (*n=12*; Control), and GII (*n=1 2*; Multiple stresses). GI ewes were maintained in the shed, while GII ewes were subjected to multiple stresses (thermal, nutritional, and walking stress). The estrus % differed significantly (*P< 0.05*) between the groups. Estrus duration also showed similar trend to that of estrus %. Both conception and lambing rate also differed significantly (*P< 0.05*) between the control and multiple-stress group. In addition plasma estradiol and progesterone also showed significant difference between the groups. The study proved the detrimental effects of multiple-stresses on various reproductive parameters studied. Hence it is very pertinent to conclude that when two or more than two stressors occur simultaneously, the total impact may be severe on reproductive functions of the animals.

## 1. Introduction

A major proportion of small ruminant population (>70%) thrive well in arid and semiarid areas of western region and southern peninsular region of India. The key constraints in arid and semiarid tropical environment are their low biomass productivity, high climatic variability, and scarcity of water. All these constraints make these regions difficult for sustainable livestock production. In particular, the productive potential of sheep and goat in these areas is influenced by the exposure to harsh climatic conditions, namely, high ambient temperature and long distance walking in search of food resources and water restriction. In the changing scenario of climate change, thermal stress along with feed and water scarcity is the major predisposing factors for the low productivity of small ruminants under hot semiarid environment. 

Sheep in hot semiarid environment are reared in extensive system. The productive potential of sheep in these areas is influenced by the exposure to harsh climatic factors [1]. Sheep grazing in this ecological zone face extreme fluctuations in the quantity and quality of feed on offer year round [2]. Despite the general awareness that energy demands vary between different seasons, information on how thermal stress influence biological functions when coupled with long term nutritional stress in sheep is scarce. Further, much still needs to be learned on the effect of simultaneous multiple stresses on livestock, which is a common occurrence in hot arid and semiarid environments. In addition to thermal stress and feed scarcity, the animals need to walk long distances for grazing in this ecological zone [3, 4]. Hence apart from heat stress, and feed and water scarcity, the animals are exposed to physical stress while walking in hot semiarid environment. These stresses lead to alteration in the process of homeostasis and metabolism. Therefore, it is importance to study the influence of all these three stresses simultaneously in order to understand the adaptive capability of sheep and identify the ideal requirements for sheep to counteract such environmental extremes. 

Studies investigating the effect of stress on farm animal productivity have generally focused on single stress at a time. Limited data are available in the literature on studies targeting the effects of multiple stresses in farm animals. Hence, the present study has been undertaken to study the influence of more than one stress simultaneously on the reproductive performance of sheep. The hypothesis of the present study is that multiple stresses effecting livestock productivity is a common phenomenon due to climate change under semiarid tropical environment. With these views, the present paper has been undertaken with the primary objective to study the effect of multiple stresses on the reproductive performance of Malpura ewes.

## 2. Materials and Method

### 2.1. Location and Climatology

The experiment was carried out at the Central Sheep and Wool Research Institute farm (Avikanagar, Rajasthan), which is located in the semiarid region of India at longitude 75°28^′  ^ E and the latitude of 26°26′ N and at altitude of 320 m above mean sea level. The average annual maximum and minimum ambient temperature ranges between 6 and 46°C. The mean annual relative humidity ranges between 20 and 85%. The annual rainfall in this area ranges from 200 to 400 mm with an erratic distribution throughout the year. The experiment was carried out during April to May. The mean environmental temperatures, relative humidity, wind velocity, and temperature-humidity index (THI) during the study period (35 days) are depicted in Table 1. THI was calculated by the formula as described by Marai et al. [5]. 

### 2.2. Animals

The study was conducted in twenty four adult (2–4 years old) Malpura nonpregnant ewes weighing between 25 to 32 kg. The animals were housed in well-ventilated sheds with asbestos roofing at 2.4 m. The sheds were open from sides and the sheds were also maintained under proper hygienic conditions. Ewes in the shed were exposed to a temperature range of 35–41°C (Average 38°C) and relative humidity (RH) of 15–20%. The animals were provided with good quality drinking water. Prophylactic measures against sheep diseases like sheep pox, peste des petits ruminants, enterotoxaemia, and endo and ectoparasitic infestations were carried out as prescribed by the health calendar of the institute to ensure that the animals were in healthy condition throughout the study.

### 2.3. Experimental Procedure

The present study was conducted for a period of 35 days covering two estrous cycles during summer season (April-May). The ewes were randomly allocated into two groups of twelve animals each namely, GI (*n* = 12; Control), and GII (*n* = 12; Multiple stresses). Care was taken while grouping the animals to ensure that there is no significant changes between the groups on average body weight before start of experiment. The animals were stall fed with a diet consisting of 70% roughage (*Cenchrus ciliaris*) and 30% concentrate (barley, 650 g/kg, groundnut cake, 320 g/kg, minerals 30 g/kg including 10 g/kg NaCl, with crude protein = 180 g/kg and total digestible nutrients = 650 g/kg). Both GI and GII ewes were stall fed at 7 : 00 h and had access to feed and water up to 9 : 00 h. After this, both groups had access to feed and water from 17 : 00 h to 16 : 30 h. This ensures uniform accessibility of both groups to feed and water. GI ewes were maintained in the shed, while GII ewes were subjected to multiple stresses. As the experiment was conducted during extreme summer months, the average temperature was at 42°C which imposed the heat stress on these animals. GI ewes were provided with *ad libitum* feeding, while GII ewes were provided with restricted feed (30% of intake of GI ewes) to induce nutritional stress. Further, GII ewes were subjected to walking stress by allowing them to walk for 14 km in two spans between 9 : 00 h to 15 : 00 h. The ewes subjected to walking stress (GII) were prevented from grazing by applying a face mask made up of cotton thread to prevent the animals grazing and to maintain the feeding pattern as per the experimental protocol. All ewes were synchronized for estrus at the start of the study using indigenously developed intravaginal sponges [6]. At the end of the study, all ewes that exhibited estrus response was mated with rams of proven vigor. 2 mL of blood were collected at weekly intervals from both groups simultaneously at 11 : 00 h using 20 gauge sterilized needles and plastic syringe from external jugular vein in tubes with heparin anticoagulant. Blood, in GII animals, was collected after completing the first span of walking to ensure effect of walking stress is established on the parameters studied. Blood samples were divided in two aliquots. Plasma was separated from blood by centrifugation at 3,500 rpm at room temperature for 20 minutes. The plasma was divided into aliquots in microcentrifuge tubes and kept frozen at −20°C till further analysis. The parameters studied were estrus %, estrus duration, conception rate, lambing rate, birth weight of lambs, plasma estradiol, and progesterone. The study was conducted after obtaining approval from the institute ethical committee for subjecting the animals to multiple stresses. 

### 2.4. Estrus Synchronization and Mating

Estrus synchronization was carried out in all the animals using indigenously developed intravaginal sponges impregnated with progesterone [6]. Each vaginal sponge was imbibed with 0.35 gm progesterone (CDH Laboratory reagent, New Delhi). The sponge was inserted and kept *in situ* in the vagina for a period of 12 days. On the day of sponge removal, ewes were given a single dose of equine chorionic gonadotropin (Folligon, Intervet International, Netherland) 200 IU/ewe intramuscularly. Estrus in each ewe was detected by parading aproned rams of proven vigor at every 6 h intervals for 30 min at early morning (6 : 00 h), noon (12 : 00 h), evening (18 : 00 h), and midnight (24 : 00 h). The ewes in estrus at the end of second cycle were hand mated twice, at 12 hrs after onset of estrus and 12 hrs later with a ram of proven fertility, usually in the morning and evening. 

### 2.5. Hormone Analysis

Plasma estradiol and progesterone concentrations were estimated by radio immunoassay (RIA) using gamma counter (PC-RIA MAS, Stretec, Germany). Estradiol in blood plasma was determined by a validated RIA kit supplied by Immunotech, France. The intra-assay and interassay coefficient of variations were 5.8% and 9.0%, respectively. The assay sensitivity for estradiol was 6 pg/mL. Progesterone concentration in blood plasma was measured by RIA kit supplied by the same Immunotech, France. The intra-assay and interassay coefficient of variations were 12.1% and 11.2%, respectively. The analytical sensitivity for plasma progesterone was 0.05 ng/mL. Estradiol and progesterone RIA assays were validated for parallelism and recovery for ewes in our laboratory as described by Kiyma et al. [7]. The antibody used in the either immunoassay was highly specific for both estradiol and progesterone, respectively. For dilution test, the recovery percentages obtained were between 83% and 108% for estradiol, while for progesterone the recovery percentage ranged between 87% and 115%. In recovery test, the recovery percentages obtained were between 82% and 111% for estradiol, while for progesterone it is between 85% and 110%. The measurement ranges were between 6–5000 pg/mL for estradiol and between 0.05–60 ng/mL for progesterone. 

### 2.6. Statistical Analysis

Differences in estrus response, conception rate and lambing rate, were evaluated by Chi-square test [8]. Differences among groups for estrus duration, estrus interval, and birth weight were evaluated by analysis of variance. Repeated data for parameters including body weight, plasma estradiol, and progesterone were analyzed using GLM procedure by analysis of variance for repeated measurements using SPSS software, version 10.0. 

## 3. Results

### 3.1. Climatological Data

The average daily climatological variables are depicted in Table 1. These recorded data suggests that the animals were under extreme stress condition throughout the study as per the THI index described by Marai et al., [5]. 

### 3.2. Reproductive Performance

The estrus % differed significantly (*P* < 0.05) between the groups (Table 2). The lowest value was recorded in group 2 as compared to group 1. Estrus duration also showed similar trend to that of estrus % with the significant (*P* < 0.05) lowering of duration being in Group 2 as compared to group 1 (Table 2). The present study also revealed that conception rate was significantly (*P* < 0.05) lower in multiple-stress group (Table 2). The same trend as that of conception rate was recorded in lambing rate also. Laming rate recorded in multiple stresses group was significantly (*P* < 0.05) lower than control group (Table 2). However, birth weight of lambs did not differed significantly between the groups. 

### 3.3. Reproductive Hormone Levels

**Figure 1:**
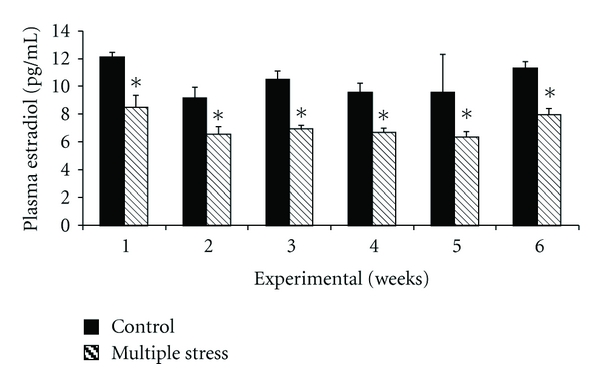
Effect of multiple stresses (thermal, nutritional, and walking stress) on the plasma estradiol levels in Malpura ewes. *Indicates statistical significance to the respective control values at *P* < 0.05.

**Figure 2:**
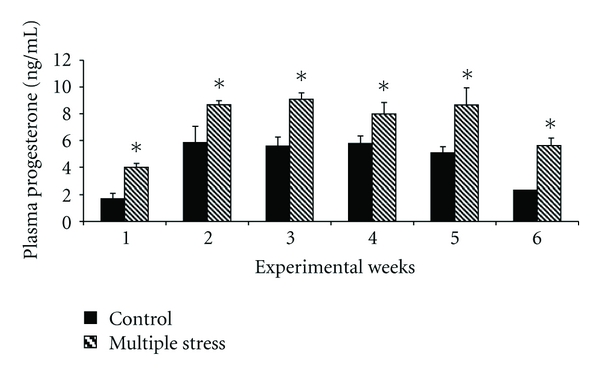
Effect of multiple stresses (thermal, nutritional, and walking stress) on the plasma progesterone levels in Malpura ewes. *Indicates statistical significance to the respective control values at *P* < 0.05.

The effect of multiple stresses was established on both plasma estradiol and progesterone level.  Figure 1 illustrates the effects of multiple stresses on plasma estradiol level. Plasma estradiol reduced significantly (*P* < 0.05) in multiple-stress group as compared to control group. This trend was consistent on all weeks of the study. However, plasma progesterone showed reverse trend for the treatment as compared to plasma estradiol.  Figure 2 illustrates the effects of multiple stresses on plasma progesterone level. Plasma progesterone increased significantly (*P* < 0.05) in GII as compared to GI. The decreasing trend established on plasma progesterone in multiple-stress group during first week of study was maintained on all weeks. 

## 4. Discussion

Thermal stress influence on estrus incidences is a well established fact [9, 10]. It is an established fact that reproduction processes are influenced during thermal exposure [11]. Further, there is evidence for influence of nutrition on folliculogenesis and ovulation rate [12]. Inadequate nutrition delays or prevents the onset of puberty, interferes with normal cyclicity in the female, and results in hypogonadism [13]. This in turn affects all other reproductive processes. Further, it is an established fact that walking stress also influences reproductive performance of sheep [4]. 

The present study revealed that conception rate was significantly (*P* < 0.05) lower in multiple stresses group. The probable reason for this could be reduced sex steroid receptor and altered sex steroids concentration due to thermal, nutritional, and walking stress in this group [4]. This view is supported by the fact that there is evidence for inhibitory effect of undernutrition on the number of uterine sex steroid receptors, which will in turn affect conception rate [14]. Lambing rate also showed results similar to conception rate in the present study. The probable reason for low lambing rate in GII could be insufficient progesterone concentration in these ewes to maintain pregnancy as the animals resumed *ad libitum* feeding after mating at the end of the experimental period. Higher level of nutrition has been found to be associated with lower circulating progesterone concentrations in ewes [15]. However, the birth weight of lambs did not differ significantly between groups.

 Plasma estradiol and progesterone showed reverse trend for the treatment. Plasma estradiol decreased significantly (*P* < 0.05), while plasma progesterone increased significantly (*P* < 0.05) in GII as compared to GI. This finding was similar to the one established in the same laboratory involving heat and nutritional stress simultaneously in the same breed [1]. Endocrine responses to stress in general work towards suppressing productive functions such as growth and reproduction, in favour of maintenance and survival [16–18]. It is generally accepted that nutrition modulates reproductive endocrine function in many species including sheep [19]. Decreased concentration of estrogen may result from diminished ovarian follicular development caused by suppressed peripheral concentration of gonadotrophins [20]. Further, as compared to the combined stress (thermal and nutritional stress) study [1], the level of plasma estradiol was much lower in this study after exposing them to multiple stresses. This reduced estradiol level could be attributed to the addition stress caused by walking stress in the present study. The level of progesterone was significantly higher in GII as compared to GI. Stress-induced increase in plasma progesterone is an established fact [1]. The level of nutrition and peripheral progesterone concentrations are inversely related [21, 22] in ewes. This inverse relationship between level of feed intake and plasma progesterone concentration was attributed to difference in metabolic clearance rate of progesterone [23]. Forcada and Abecia [24] findings of difference in the rate of clearance rather than differences in secretion levels can explain the apparent inverse relationship between nutrition and peripheral progesterone concentrations in ewes. 

## 5. Conclusions

The study proved the detrimental effects of multiple stresses on various reproductive parameters studied in Malpura ewes. Hence it is very pertinent to conclude that when two or more than two stressors occur simultaneously, the total impact may be severe on the reproductive functions. 

## Figures and Tables

**Table 1:** Mean and SEM of meteorological data during the study period.

Weather parameters	Daily average
Minimum temperature (°C)	37.59 ± 0.32
Maximum temperature (°C)	38.64 ± 0.36
Dry bulb temperature (°C)	38.76 ± 0.36
Wet bulb temperature (°C)	21.31 ± 0.24
Relative humidity (%)	17.33 ± 0.75
Temperature humidity index	32.48 ± 0.26

The values are the averages of 35 days study period.

**Table 2:** Effect of multiple stresses (thermal, nutritional, and walking stress) on reproductive parameters in Malpura ewes.

Reproductive parameter	GI	GII
Estrus %	66.67 (8/12)^a^	41.7 (5/12)^b^
Estrus duration (hrs)	32^a^	14.4^b^
Conception rate (%)	83.3 (10/12)^a^	50 (6/12)^b^
Lambing rate (%)	83.3 (10/12)^a^	50 (6/12)^b^
Birth weight of lambs (kg)	3.35 ± 0.14^a^	3.60 ± 0.10^a^

Means and SE with different superscripts in same row differ significantly *P* < 0.05.
